# The Brown Sugar Mediated Carbon Quantum Dots as a Novel Fluorescence Sensor for Sensitive Detection of Gentamicin and Its Application in Foods

**DOI:** 10.3390/ijms25042143

**Published:** 2024-02-10

**Authors:** Xinran Guo, Yanxin Guo, Xinyue Chen

**Affiliations:** Institute of Pharmaceutical Analysis, School of Pharmacy, Lanzhou University, Lanzhou 730030, China; guoxr21@lzu.edu.cn (X.G.); gyx4847614@163.com (Y.G.)

**Keywords:** carbon quantum dots, gold nanoparticles, green synthesis, fluorescence probe, gentamicin

## Abstract

In this work, a novel fluorescence sensing strategy was proposed for the detection of gentamicin based on fluorescent carbon quantum dots (CQDs) and gold nanoparticles (AuNPs). Herein, the CQDs were green-synthesized for the first time via a one-step hydrothermal method utilizing brown sugar as the precursor. In the presence of citrate-stabilized AuNPs, the fluorescence of CQDs was quenched efficiently. Gentamicin, on the other hand, had a higher affinity for AuNPs and was able to compete with CQDs for a preferential binding to AuNPs, which ultimately led to the aggregation of AuNPs and freeing of CQDs in solution, causing the fluorescence recovery of CQDs. Based on the above phenomenon, the concentrations of gentamicin could be ascertained by detecting the variations in fluorescence intensity of CQDs. This sensing strategy exhibited excellent selectivity in various antibiotics. At the same time, the method displayed outstanding sensitivity for gentamicin, which was successfully applied to real samples detection.

## 1. Introduction

Gentamicin, as a kind of broad-spectrum aminoglycoside antibiotic, inhibits protein synthesis by acting on the ribosomes of pathogenic bacteria, thus exerting antibacterial effects. Gentamicin is widely used in the treatment of bacterial infections especially caused by Gram-negative bacteria [1]. Gentamicin is often heavily utilized to prevent and treat infections in domestic animals in animal husbandry and agricultural production. As a result, gentamicin will remain in some agricultural products such as milk, eggs and meat and eventually be absorbed into the human body [2]. Gentamicin is known to be nephrotoxic and ototoxic due to the ability of its metabolites to interact with the cochlea and renal cortex [3,4]. In addition, it has been demonstrated that the widespread use of gentamicin may lead to a further development of bacterial resistance [5]. The European Commission has stipulated that the maximum residue limit (MRL) of gentamicin is 100 μg kg^−1^ (173.7 nM) in milk in Europe [6]. Therefore, residual gentamicin in food and the environment has adverse effects on human health safety. It is critical to monitor gentamicin levels in the environment and in food products. Currently, the quantitative detection methods of gentamicin include high-performance liquid chromatography (HPLC) [7,8], liquid chromatography-tandem mass spectrometry (LC-MS) [9], chromatography–mass spectrometry (GC-MS) [10], electrochemical [11] and immunoassays [12,13]. Most of these assays require complex sample derivatization, expensive instruments, long analysis times and complicated procedures. Therefore, it is necessary to develop a simple, low-cost, and rapid assay for the detection of gentamicin.

In recent years, CQDs (carbon quantum dots), as an emerging zero-dimensional carbon nanomaterial, have attracted much attention in the field of constructing nanosensors because of their simple synthesis method, low-cost synthesis, outstanding optical properties, and good biocompatibility [14,15]. Moreover, due to the above advantages, CQDs are also widely used in biologically relevant applications such as drug delivery, bioimaging and photothermal therapy [16,17,18,19]. The most common method for synthesizing CQDs is the one-step hydrothermal method by using readily available and inexpensive biomass as a carbon source in daily life without the use of toxic small molecule organic precursors [20,21]. Compared with traditional inorganic quantum dots and organic dyes, CQDs through green synthesis have properties such as lower toxicity and good water solubility, which further extends the application scope of CQDs [15,22]. On the other hand, gold nanoparticles (AuNPs) have a high fluorescence quenching efficiency due to their wide absorption spectra and usually act as fluorescence quenchers [23]. According to this feature, it is easy to find the reported studies of biochemical fluorescent sensors based on CQDs and AuNPs. For example, Li et al. developed a sensitive and selective nanosensor to detect GSH using N and S-CDs/AuNPs in human serum [24]. Fu et al. proposed a signal “on–off–on” fluorescent nanosensor based on AuNPs and N-CDs for the accurate qualitative and quantitative measurement of pesticide thiram in hawthorn [25]. Wang et al. synthesized novel N, C-dots and assembled them on aptamer/AuNPs for the super-sensitive detection of aflatoxin B1 [26]. The above studies suggest that the development of fluorescent sensors based on CQDs and AuNPs is very promising for research and applications.

**Figure 1:**
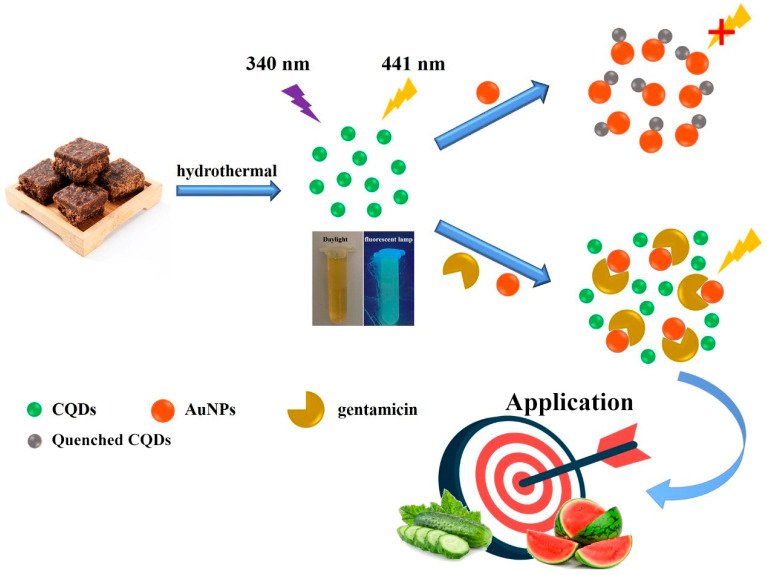
The diagram illustration of the principle for the sensitive detection of gentamicin based on brown sugar-derived CQDs and AuNPs, which was applied in watermelon and cucumber samples.

In this work, CQDs were rapidly synthesized via a one-step hydrothermal method using commercially available brown sugar as the carbon source (Figure 1). The surface structure, morphology, and optical properties of CQDs were studied through various characterization methods. The synthesized CQDs had good water solubility and excellent photoluminescence performance with the maintenance of stable fluorescence emission under extreme conditions. AuNPs were obtained by using a common sodium citrate reduction method. AuNPs have strong fluorescence quenching efficiency due to their large molar extinction coefficient, and their wide UV absorption spectrum could overlap with the fluorescence excitation and emission spectrum of CQDs [23,27]. Therefore, the fluorescence of CQDs was effectively quenched after the addition of AuNPs. However, when gentamicin was added, it could bind tightly with AuNPs through the interaction of Au-N bonds due to gentamicin having five aliphatic amino groups on its structure [28]. The preferential binding of gentamicin and AuNPs caused the aggregation of AuNPs and the release of CQDs. As a result, the fluorescence recovery of the system could be clearly observed. By evaluating the recovered fluorescence intensity, the concentration of gentamicin could be quantitatively assessed. Thus, a novel fluorescent “on–off–on” method for the detection of gentamicin was proposed in this work. This method had the advantages of low cost, simplicity, rapidity, good sensitivity and selectivity, and it had been well applied in the testing of real samples.

## 2. Results and Discussion

### 2.1. Characterization of CQDs

**Figure 2:**
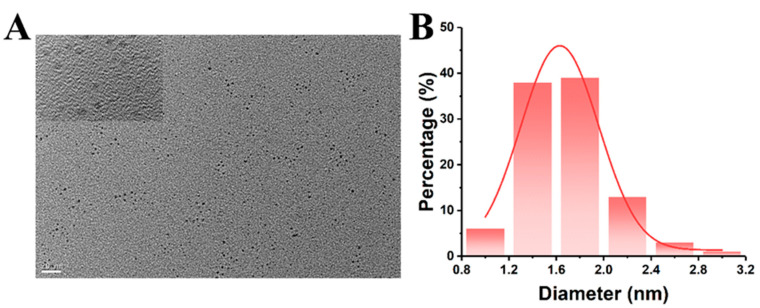
(**A**) TEM image (20 nm) and HR-TEM image (2 nm, inset) of CQDs; (**B**) size distribution histograms of CQDs.

**Figure 3:**
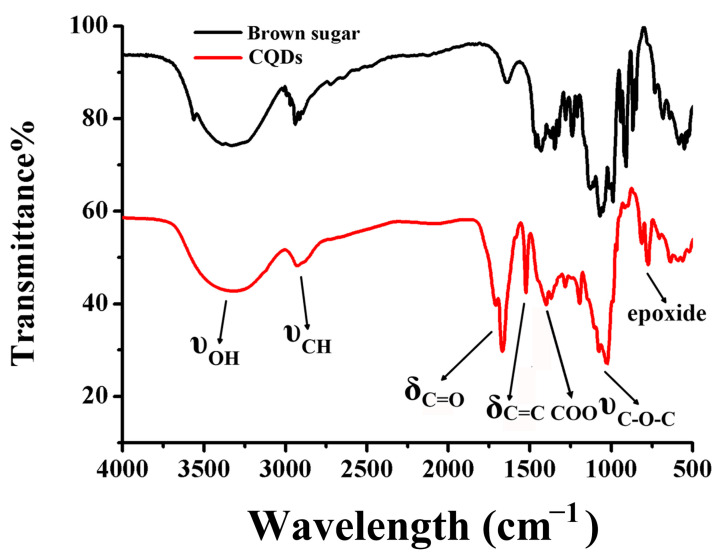
FTIR spectra of brown sugar (black line) and CQDs (red line).

Multiple analysis methods were carried out to demonstrate the morphological appearance, elemental and surface group composition of the prepared CQDs. First of all, the size distribution and morphology of CQDs were evaluated by TEM. As shown in Figure 2A, the prepared CQDs were spherical in shape and well dispersed in the solution. It was discovered that the particle size distribution of CQDs was 1.09–3.06 nm with an average size of 1.68 nm, as displayed in Figure 2B. FITR spectroscopy was utilized to analyze the molecular functional groups of CQDs. As shown in Figure 3, four new absorption peaks appeared at 1668 cm^−1^, 1523 cm^−1^, 1399 cm^−1^ and 772 cm^−1^ that could be ascribed to the C=O band, C=C band, COO band and epoxide bending vibration [29,30]. The bands at 3324 cm^−1^ and 2928 cm^−1^ could be attributed to the stretching vibration of O-H and C-H [31,32], respectively. In addition, the peak at 1027 cm^−1^ was assigned to C-O-C [33]. Compared with the FTIR result of the precursor brown sugar, the absorption peaks were in essentially the same position and hardly showed any shift. Abundant functional groups were obtained after hydrothermal treatment, suggesting that the surface of CQDs was covered by various oxygen functional groups.

**Figure 4:**
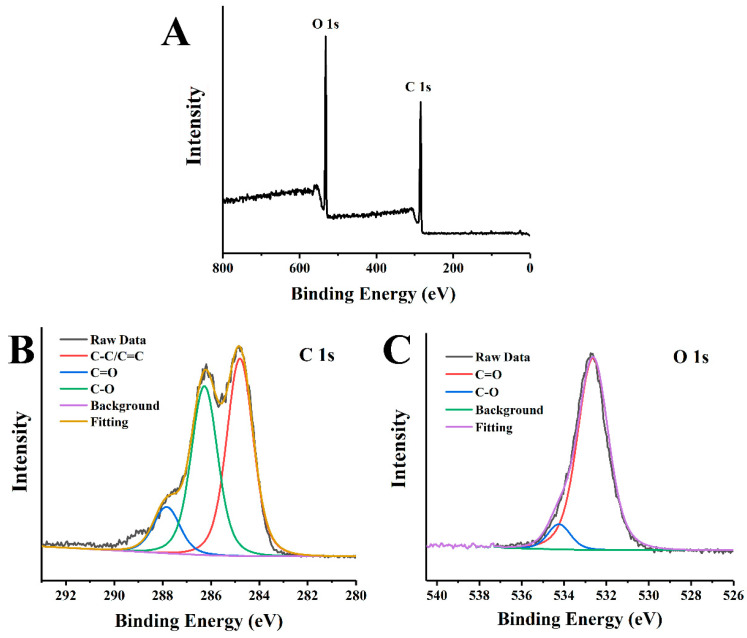
(**A**) Full XPS spectra of CQDs and high-resolution XPS spectra of (**B**) C 1s and (**C**) O 1s.

**Figure 5:**
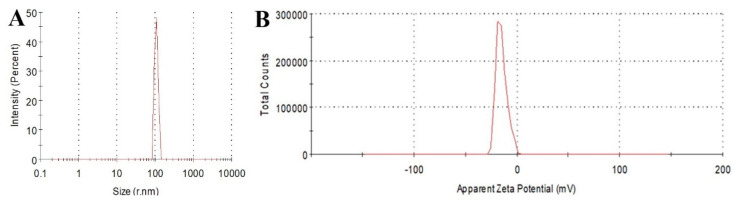
(**A**) The particle size distribution and (**B**) zeta potential of CQDs.

Furthermore, the XPS analysis was performed to characterize the elemental composition and surface functional groups of CQDs. In the full-range XPS (Figure 4A) spectra, there were two typical peaks at 284.8 eV and 532.6 eV attributed to C 1s and O 1s, respectively, and the corresponding atomic contents were 73.56% (C) and 26.44% (O). The high-resolution C 1s spectra (Figure 4B) illustrated three characteristic peaks of C-C/C=C, C-O and C=O at 284.8 eV, 287.8 eV and 286.3 eV, respectively [34,35]. Moreover, the two peaks at 532.7 eV and 534.3 eV in the O1s spectra (Figure 4C) could be assigned to C=O and C-O bands [36]. DLS results revealed that the hydrated particle size of CQDs (Figure 5A) was over 100 nm. The larger size result of the nanoparticles was obtained through dynamic light scattering, which might be due the solvation effect around the CQDs. Because CQDs had hydrophilicity and high specific surface area, polar molecules in solution preferred to attach to them [37]. The surface potential of CQDs (Figure 5B) was negative with a zeta potential of −20 mV. 

In summary, after analyzing the CQDs by various characterization methods, it could be revealed that the CQDs were nearly spherical in shape, and the surface was rich in hydroxyl, carboxyl and carbonyl groups. Moreover, these results were consistent with what had been reported in the literature [38,39].

**Figure 6:**
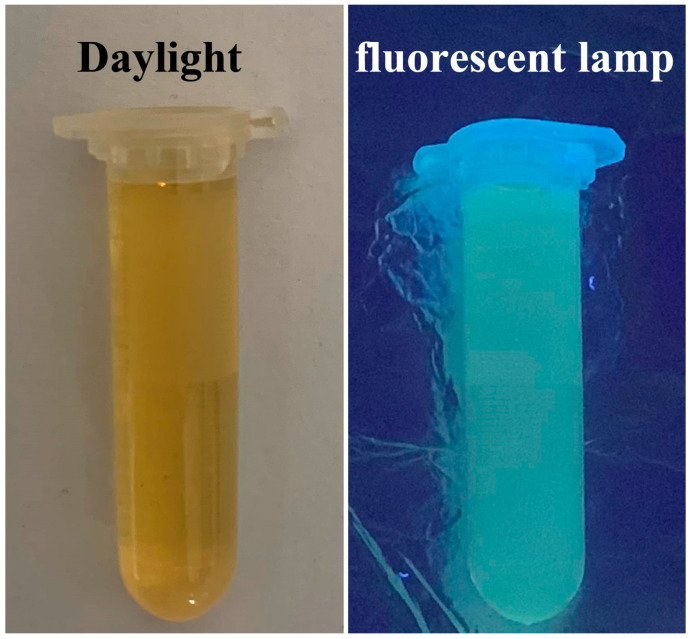
Photographs of CQDs solution under sunlight (**left**) and 365 nm UV light (**right**).

**Figure 7:**
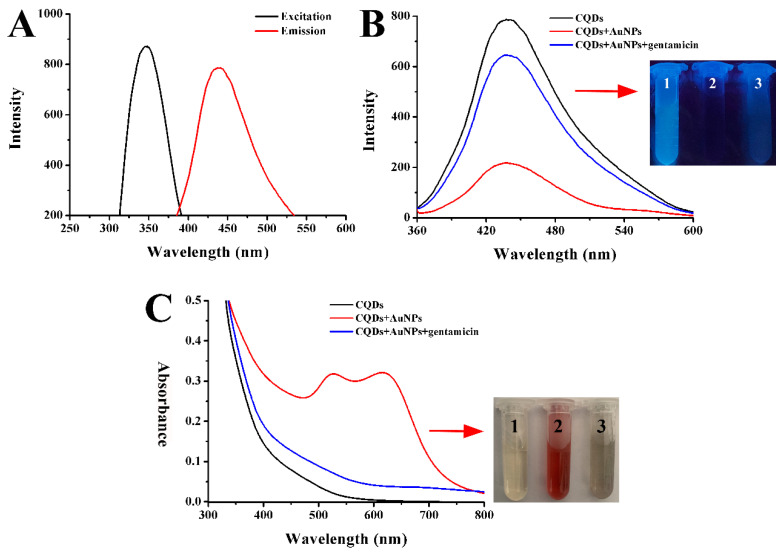
(**A**) Fluorescence excitation and emission spectra of CQDs. (**B**) Fluorescence spectra of CQDs, CQDs + AuNPs, CQDs + AuNPs + gentamicin. The inset was the photograph of CQDs (1), CQDs + AuNPs (2) and CQDs + AuNPs + gentamicin (3) under a UV lamp. (**C**) UV-Vis absorption spectra of CQDs, CQDs + AuNPs, CQDs + AuNPs + gentamicin. The inset was the photograph of CQDs (1), CQDs + AuNPs (2), CQDs + AuNPs + gentamicin (3) under visible light.

As illustrated in Figure 6A,B, the CQDs solution appeared light brown under visible light and emitted blue-green fluorescence under 365 nm UV light, and the concentration of the aqueous CQDs solution was 10.55 mg/mL. Figure 7A shows the maximum fluorescence excitation and emission spectra of CQDs. When the excitation light was 350 nm, the corresponding maximum emission wavelength was 440 nm.

### 2.2. Stability Test of CQDs

**Figure 8:**
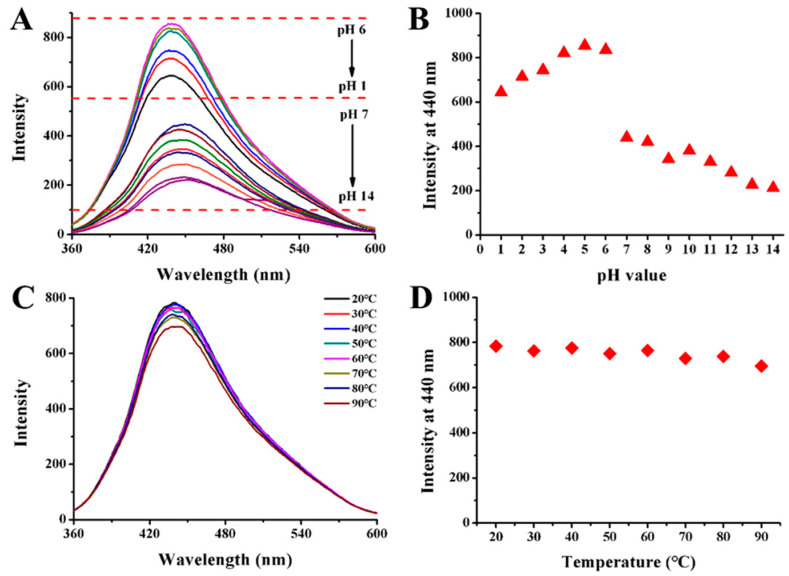
The fluorescence spectra of CQDs solution with pH variations from (**A**) 1 to 14 and (**C**) temperature variations from 20 to 90 °C. Trends of fluorescence intensity at 440 nm when (**B**) pH changed from 1 to 14 and (**D**) temperature changed from 20 to 90 °C.

The fluorescence stability of the prepared CQDs in a variety of conditions was evaluated. As illustrated in Figure 8A,B, the influence of pH value was investigated in a range of 1–14. The initial pH of the CQDs solution was 7, and the fluorescence intensity of the CQDs was increased when the solution pH was adjusted to acidic. This may be due to the increased protonation of the surface groups of CQDs under acidic conditions [40]. When the CQDs solution was under strong acid or strong alkaline conditions, the fluorescence intensity could be maintained in a relatively stable state, indicating the stable property of CQDs to pH. Moreover, the effect of temperature on the fluorescence of CQDs was studied (Figure 8C,D). With the solution heating from 20 to 90 °C, the fluorescence intensity scarcely changed, suggesting CQDs had a good high-temperature resistance.

### 2.3. Fluorescence Detection of Gentamicin Based on CQDs and AuNPs

**Figure 9:**
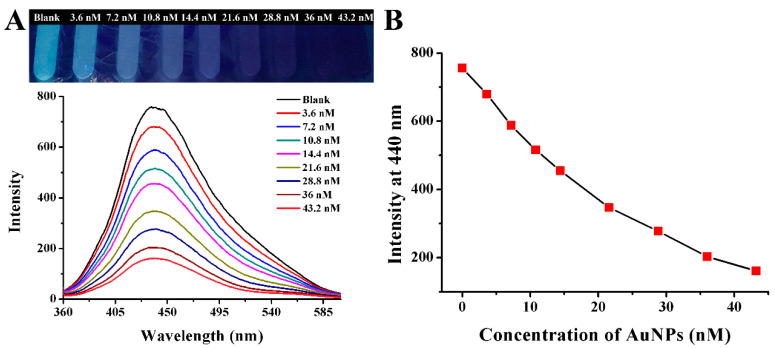
(**A**) Photograph and fluorescence spectra of CQDs with addition of different concentrations of AuNPs (final concentration). (**B**) The variation relationship between different concentrations of AuNPs and fluorescence intensity at 440 nm of CQDs.

AuNPs have attracted a lot of attention because of their high extinction coefficient [41] and broad absorption spectrum in a visible light that overlaps with the emission wavelength of typical energy donors. It had been demonstrated that AuNPs have a great quenching efficiency [42,43]. Therefore, the quenching efficiency of AuNPs toward CQDs was investigated. As shown in Figure 9, AuNPs had a superior quenching effect on CQDs. The blue fluorescence of CQDs gradually decreased (Figure 9A) when the concentration of AuNPs increased from 0 to 43.2 nM. And the fluorescence intensity at 440 nm decreased significantly with the concentration of AuNPs increasing correspondingly (Figure 8B). Finally, 43.2 nM of AuNPs was chosen as the optimal concentration for quenching the fluorescence of CQDs. 

As mentioned above, AuNPs could quench the fluorescence of CQDs effectively. Interestingly, as shown in Figure 7B, when gentamicin was added to the AuNPs-CQDs system solution, the blue fluorescence of CQDs was restored and the fluorescence intensity increased significantly. As depicted in the UV-vis spectra of Figure 7C, a distinct absorption band could be observed at 500 to 700 nm after AuNPs were added to the CQDs solution, which could be ascribed to the strong absorption peak of the AuNPs. The insert image in Figure 7C showed the dispersed AuNPs with a wine-red color, indicating that the obvious agglomeration of AuNPs did not occur when mixed with CQDs. After the addition of gentamicin to the AuNPs and CQDs solution, the SPR peak belonging to AuNPs disappeared along with the appearance of obvious gray–black precipitates in the solution. In addition, previous studies reported that metal ions have a good bursting effect on CQDs [44]. After investigation, Fe^3+^ was revealed to have the ability to quench the fluorescence of CQDs. As illustrated in Figure S1, the fluorescence intensity of CQDs gradually decreased as the concentration of Fe^3+^ increased. However, the fluorescence of CQDs was completely quenched when the concentration of Fe^3+^ reached 5.6 mM. AuNPs could completely quench CQDs fluorescence with as little as 43.2 nM, which had better fluorescence quench ability. As shown in Figure S2, the addition of various concentrations of gentamicin to the CQDs-Fe^3+^ system failed to recover the fluorescence, which was attributed to the fact that Fe^3+^ did not have a good affinity for gentamicin. The fluorescence of CQDs did not recover. Therefore, the AuNPs were chosen as the optimal quencher because of their great fluorescence quench efficiency and strong binding ability with the detection of the target, gentamicin.

**Figure 10:**
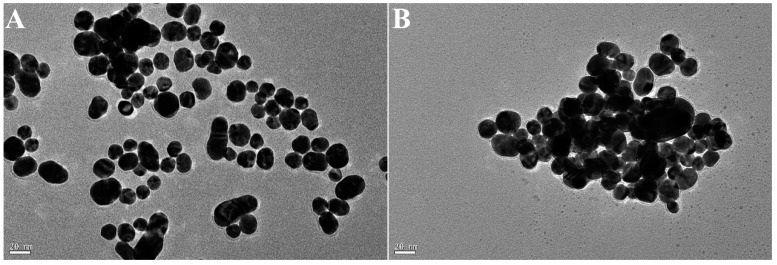
The TEM images of CQDs + AuNPs (**A**) and CQDs + AuNPs + gentamicin (**B**).

The TEM images of CQDs and AuNPs solution before and after the addition of gentamicin are shown in Figure 10. Prior to the addition of gentamicin, AuNPs underwent slight aggregation due to the interaction with CQDs (Figure 10A), which is accompanied by the fluorescence quenching of CQDs. After the further addition of gentamicin, AuNPs significantly agglomerated, while the small particles appearing around in Figure 10B indicated dispersed CQDs in solution. This occurred perhaps because gentamicin was an aminoglycoside antibiotic with five amino groups in its structure. The tight binding between gentamicin and AuNPs via interactions of Au-N bonds induced the aggregation of AuNPs and the freeing of CQDs, eventually leading to the recovery of fluorescence of CQDs. The changes in the TEM images also confirmed the corresponding color changes in Figure 9. The above results illustrate the feasibility of the sensing strategy, based on which a fluorescence detection method for gentamicin was proposed.

**Figure 11:**
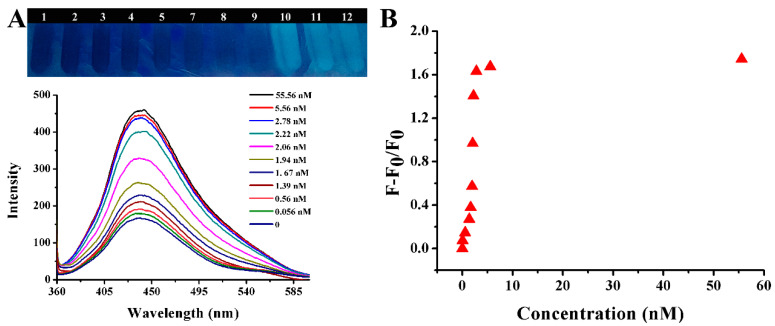
(**A**) Photograph and fluorescence spectra of CQDs and AuNPs solution after adding different concentrations of gentamicin. (**B**) A dynamic response between gentamicin concentration and (F − F_0_)/F_0_ at 440 nm.

The fluorescence spectra of the solution in the presence of different concentration of gentamicin were manifested in Figure 11. As shown in the Figure 11A, the fluorescence intensity of CQDs at 440 nm was gradually increased, and blue fluorescence gradually became apparent as the concentration of gentamicin increased. The fluorescence recovery efficiency (F − F_0_)/F_0_ continued to increase as the concentration of gentamicin increased, as shown in Figure 11B. The trend of change could be clearly observed. This proved that gentamicin had a strong ability to recover the fluorescence of the CQDs-AuNPs system once again.

### 2.4. Selectivity Study

**Figure 12:**
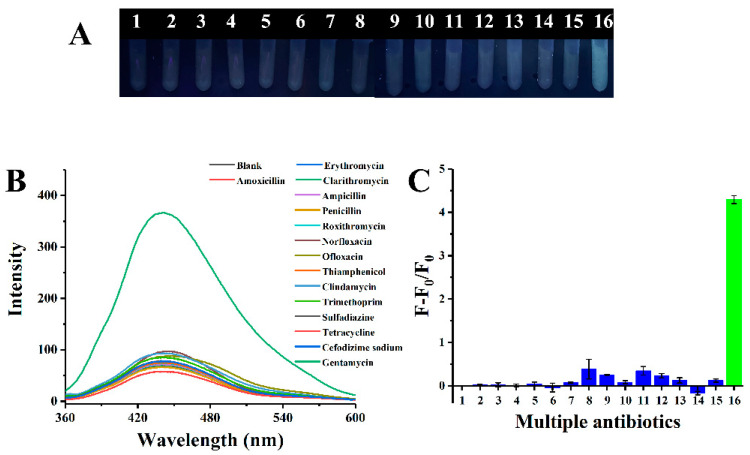
(**A**) Photograph and (**B**) fluorescence spectra of CQDs and AuNPs solution after addition of various antibiotics. (**C**) The histogram corresponds to (**B**). 1–16 represent, respectively, blank, amoxicillin, erythromycin, clarithromycin, ampicillin, penicillin, roxithromycin, norfloxacin, ofloxacin, thiamphenicol, clindamycin, trimethoprim, sulfapyridine, tetracycline, cefodizime sodium and gentamicin. The concentrations of norfloxacin, ofloxacin, and methotrexate were 5 μM. The concentration of clindamycin was 1 μM. The concentration of gentamicin was 200 nM. The other antibiotic concentrations were 50 μM.

To demonstrate the selectivity of the proposed assay, the fluorescence response of the CQDs-AuNPs system to gentamicin was investigated in the presence of different antibiotics. Selective assays were carried out under the identical conditions using 14 common antibiotics including erythromycin, clarithromycin, ampicillin, penicillin, roxithromycin, norfloxacin, ofloxacin, thiamphenicol, clindamycin, trimethoprim, sulfapyridine, tetracycline, cefodizime sodium and amoxicillin. The as-prepared antibiotics solutions were added into the CQDs-AuNPs system, and the results are depicted in Figure 12. The gentamicin assay was not interfered with even when other antibiotics were at higher concentrations. In addition, the result of the anti-interference experiment is shown in Figure S3; coexistence with other antibiotics did not interfere with gentamicin detection. The above results suggested that the proposed detection strategy could be applied to gentamicin detection with high selectivity.

### 2.5. Determination of Gentamicin in Real Samples

**Figure 13:**
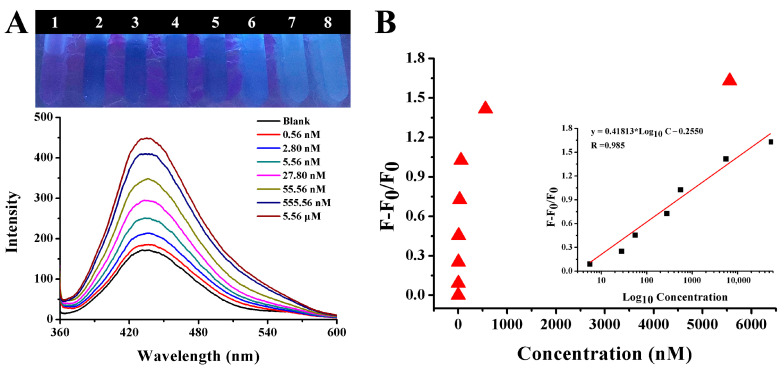
(**A**) The fluorescence spectra and photograph of CQDs and AuNPs system in the presence of increasing concentrations of gentamicin in watermelon sample. (**B**) The changing relationship between (F − F_0_)/F_0_ and different concentrations of gentamicin. Inset shows the linear calibration of (F − F_0_)/F_0_ versus the concentration of gentamicin in watermelon.

**Figure 14:**
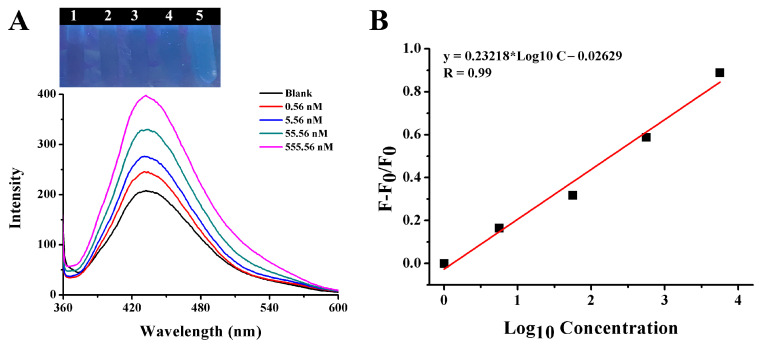
(**A**) The photograph and fluorescence spectra of CQDs and AuNPs system with an increasing concentration of gentamicin in the cucumber sample. (**B**) The linear relationship of fluorescence recovery efficiency (F − F_0_)/F_0_ versus the concentration of gentamicin.

To further verify the feasibility of the method in practical applications, different amounts of gentamicin were spiked in prepared watermelon juice and cucumber juice samples. As shown in Figure 13A,B, in the watermelon juice sample, as the concentration of gentamicin was in the range from 0.56 nM to 5.56 μM, the fluorescence intensity of CQDs-AuNPs gradually increased, and the color turned from black to blue accordingly. The correlation between the concentration of gentamicin and the fluorescence-enhanced efficiency was established as F − F_0_/F_0_ = 0.41813 × Log_10_C − 0.2550 with R = 0.985, and the LOD as seen by the naked eye was 0.56 nM. The same experiment was also conducted in the prepared cucumber juice sample, and the results are depicted in Figure 14A,B. It was clear that the fluorescence-enhanced efficiency F − F_0_/F_0_ was linear with the gentamicin concentration in the range of 0–555.56 nM, and the calibration curve could be expressed as F − F_0_/F_0_ = 0.23218 × Log_10_C − 0.02629 with R of 0.99. Table 1 shows a comparison of the linear range and LOD of different methods. This work provided a simple, rapid and sensitive method for the detection of gentamicin with a relatively wide detection range, low detection limit and high selectivity.

In addition, as shown in Figure S4, a visual colorimetric method was used to investigate the color change in the solution after the addition of gentamicin in the watermelon samples. When the concentration of gentamicin varied from 0 to 5.56 μM, the color changed from red to black. When the concentrations of gentamicin were 0.56 nM, 2.80 nM, 5.56 nM and 27.80 nM, the color of the solutions in samples 2–5 was almost the same, and the differentiation was hard to obverse. When the concentration was above 55.56 nM (samples 6–8), a large number of agglomerates were generated and precipitated in the solution. The same visual colorimetric investigation was carried out, and similar experimental results were obtained, as shown in Figure S5. When the concentrations of gentamicin were 0.56 nM, 5.56 nM and 55.56 nM in the cucumber samples, the color of the solutions (samples 2–4) shows the same deep red color. When the concentration of gentamicin was 555.56 nM, a massive agglomeration of AuNPs was used to form the gray–black precipitate. The above phenomenon indicated that the visual colorimetric method was difficult to be applied in the detection of gentamicin, which further highlighted the advantages of the fluorescence detection method.

## 3. Methods and Materials

### 3.1. Materials and Characterizations

The brown sugar was purchased from the local market. HAuCl_4_·3H_2_O was purchased from Sigma-Aldrich (St. Louis, MO, USA). Erythromycin and roxithromycin were obtained from Solarbio life sciences Technology Co., Ltd. (Beijing, China). Gentamicin, clindamycin and sulfadiazine were purchased from Yuanye Bio-Technology Co., Ltd. (Shanghai, China). The standard of clarithromycin, ampicillin, trimethoprim, cefodizime sodium and amoxicillin were purchased from Macklin Biochemical Co., Ltd. (Shanghai, China). Norfloxacin was obtained from Shanghai Aladdin Biochemical Technology Co., Ltd. (Shanghai, China). Tetracycline and thiamphenicol were purchased from Meryer Chemical Technology Co., Ltd. (Shanghai, China). All the chemical regents were analytically pure and utilized as received. Throughout the experiment, double-deionized water (DDW, A.S. Watson, Beijing, China) was utilized.

The ultraviolet absorption spectra were recorded by using ultraviolet-visible (UV-vis) spectroscopy (Perkin Elmer, lamda25, Waltham, MA, USA) in range of 300–800 nm. The fluorescent spectra were measured by using Spectro fluorophotometer (Shimadzu, RF-5301 PC, Kyoto, Japan), and the slit widths of excitation and emission light were both set as 5 nm. The FEI Tecnai G2TF20 instrument (Feitecnai, Hillsboro, OR, USA) was utilized to obtain the transmission electron microscope (TEM) image of CQDs. The FT-IR spectrometer (Nicolet, Nexus 870, Green Bay, WI, USA) was utilized to characterize the surface structure of CQDs. The X-ray Photoelectron Spectroscopy (XPS) result was investigated by using an X-ray Photoelectron Spectrometer (Shimadzu, Axis Supra, Kyoto, Japan). The size and zeta potential results were collected by a laser dynamic scattering instrument Zeta sizer Nano 3600 (Malvern, UK). 

### 3.2. Synthesis of CQDs and AuNPs

#### 3.2.1. Green Synthesis of CQDs

Through a one-step hydrothermal process, CQDs were synthesized by utilizing brown sugar as precursors for the first time. In brief, 1 g of brown sugar was dispersed in 20 mL of deionized water. After that, the mixture was placed inside a stainless-steel autoclave with Teflon lining and heated to 180 °C for three hours. After the temperature had been cooled down, the dark brown product was collected and centrifuged for 20 min at 12,000 rpm and filtered through a 0.22 μm filter membrane several times in order to remove large particles. The CQDs solution was diluted eight times and kept at 4 °C in the darkness for subsequent use and characterization finally.

#### 3.2.2. Synthesis of AuNPs

In this study, AuNPs were synthesized by reducing HAuCl_4_ with sodium citrate [49]. In brief, 1 g of HAuCl_4_·3H_2_O was dissolved in 10 mL of deionized water to obtain 10% (*w*/*v*) HAuCl_4_ standard solution. Then, 0.228 g of sodium citrate was dissolved in 10 mL of water to obtain 77.6 mM of sodium citrate solution. Afterwards, 786 μL of 10% HAuCl_4_ solution was taken and diluted to 100 mL with purified water in a 250 mL round-bottom flask, stirred, and it was heated to boiling. The 10 mL 77.6 mM sodium citrate solution was quickly poured into the boiling HAuCl_4_ solution; then, the mixture was vigorously stirred and remained boiling for 10 min. It could be clearly observed that the color of the mixed solution changed from pale yellow to wine red, suggesting the successful synthesis of AuNPs. The typical absorption wavelength of AuNPs was exhibited at 520 nm, indicating the particle size of the as-prepared AuNPs was nearly 13 nm. Lambert Beer’s law stated that A = Kbc, c = A/Kb, where A was the absorbance value, K represented the molar absorption coefficient (2.7 × 10^8^ mol^−1^ cm^−1^) [50], and b represented the thickness of the absorption layer. As a result, it was determined that the molar concentration of the as-prepared AuNPs was roughly 259 nM.

### 3.3. Fluorescence Quenching by AuNPs

First, 300 μL of CQD solution was mixed with 0, 25 μL, 50 μL, 75 μL, 100 μL, 150 μL, 200 μL, 250 μL, and 300 μL AuNPs solutions to examine the fluorescence quenching impact of various AuNP concentrations on CQDs. After that, distilled water was added to the solution to restore its entire volume at 1800 μL. AuNPs were present in the following final concentrations: 0, 3.6 nM, 7.2 nM, 10.8 nM, 14.4 nM, 21.6 nM, 28.8 nM, 36 nM, and 43.2 nM.

After a 15 s vortex, the color changes of fluorescence were observed under a UV light, and the fluorescence emission spectra were recorded under 340 nm excitation. The maximum emission wavelength was 440 nm, and the slit widths of excitation and emission light were both 5 nm. 

### 3.4. Fluorescence Detection of Gentamicin Based on CQDs and AuNPs

The 300 μL AuNPs were determined as having the optimal volume to quench the fluorescence of CQDs in subsequent studies. The process for gentamicin detection was as follows: 100 μL of different concentrations of gentamicin and 300 μL AuNPs were mixed completely by vortexing; then, 300 μL CQDs was added, and the total volume of the system was replenished to 1800 μL with distilled water. The final concentrations of gentamicin were 0.056 nM, 0.56 nM, 1.39 nM, 1.67 nM, 1.97 nM, 2.06 nM, 2.22 nM, 2.78 nM, 5.56 nM and 55.6 nM. The color changes were observed under a UV light, and the fluorescence spectrum of each sample was measured using the same settings as before.

### 3.5. Selectivity Study

It was vital to confirm the CQDs-AuNPs system’s selectivity and anti-interference property toward gentamicin in order to guarantee practical applications. In the selective experiment, 14 different antibiotics were used. The steps involved were as follows: 300 μL of AuNPs was mixed thoroughly with different antibiotics respectively, then 300 μL of CQDs and 1400 μL of distilled water were added. The anti-interference experiment was conducted by mixing gentamicin with other antibiotics. The detection method was the same as mentioned above. 

### 3.6. Detection of Gentamicin in Real Samples

Watermelon and cucumber were purchased from a local market in Chengguan District, Lanzhou, Gansu (Gansu, China). Watermelon samples and cucumber samples were processed as follows: watermelon juice was filtered using 0.22 μm membranes and diluted 10 times to obtain watermelon samples. After centrifuging cucumber juice for 10 min at 14,000 rpm, the supernatant was collected, filtered through 0.22 μm membranes, and then diluted 20 times before being used. Finally, a series concentration of gentamicin was spiked into the above samples, and the subsequent detection procedures were carried out in accordance with Section 3.4.

## 4. Conclusions

In summary, a novel “on–off–on” fluorescence detection method for gentamicin was established based on CQDs and AuNPs. Brown sugar-derived CQDs demonstrated great water dispersibility and stable fluorescence properties. The fluorescence of CQDs could be sufficiently quenched by AuNPs due to its significant molar absorption coefficient. Gentamicin competed with CQDs to bound with AuNPs tightly through stronger Au-N covalent interaction, resulting in the aggregation of AuNPs and thus leading to the release of CQDs and recovery of fluorescence. The degree of fluorescence recovery was linearly related to the concentration of gentamicin. Meanwhile, the changing trend could be clearly obtained by observing the color change in fluorescence. As a result, a novel method for the quantitative detection of gentamicin was proposed. Selective experiments indicated that multiple antibiotics did not influence the detection of gentamicin. The method had the advantages of a wide detection range and low detection limit, simple operation and low cost. The method was finally successfully applied to food samples testing.

## Figures and Tables

**Table 1:** Comparison of different methods for detection of gentamicin.

Method	Linear Range	LOD	Reference
HPLC	1.04–52.34 μM	1.04 μM	[45]
Electrochemical	0.1 μM–1 mM	75 nM	[46]
MG-AgNPs	1–100 μM	0.29 μM	[47]
Lys-AuNPs	5–60 nM	1.22 nM	[1]
Cys-AuNPs	10–200 nM	12.45 nM	[48]
CQDs-AuNPs	0.56 nM–5.56 μM	0.56 nM	This work

## Data Availability

The data presented in this study are available on request from the corresponding author.

## Supplementary Materials

The following supporting information can be downloaded at https://www.mdpi.com/article/10.3390/ijms25042143/s1.
