# Supplementary material for: The Brown Sugar Mediated Carbon Quantum Dots as a Novel Fluorescence Sensor for Sensitive Detection of Gentamicin and Its Application in Foods
Source: Int J Mol Sci. 2024 Feb 10;25(4):2143. doi: 10.3390/ijms25042143 (PMC10889699; doi:10.3390/ijms25042143)
Supplement: Supplementary file 1 [file ijms-25-02143-s001.zip › ijms-2856173-supplementary.pdf]

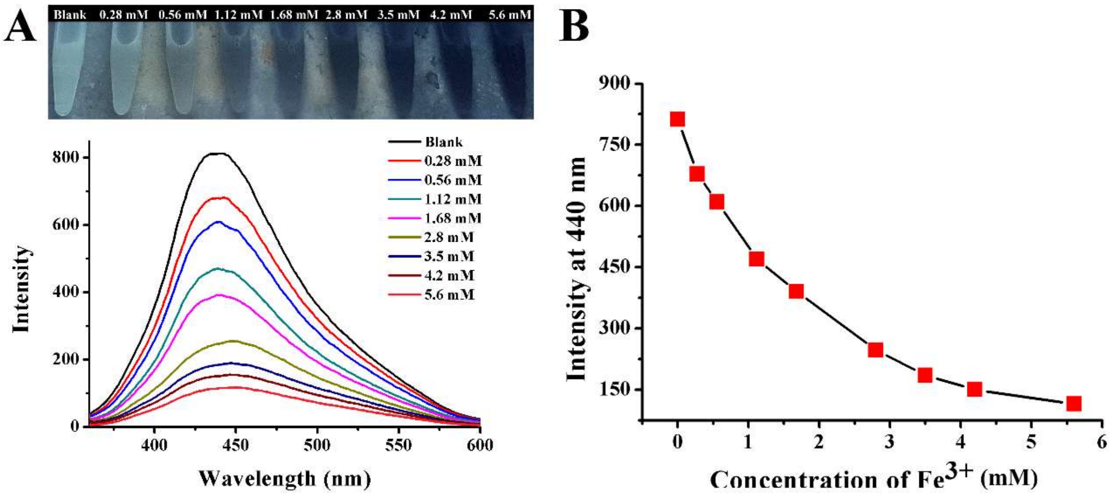

Figure S1. (**A**) Photograph and fluorescence spectra of CQDs with different concentrations of Fe^{3+}. (**B**) The variation relationship between different concentrations of Fe^{3+} and fluorescence intensity at 440 nm.

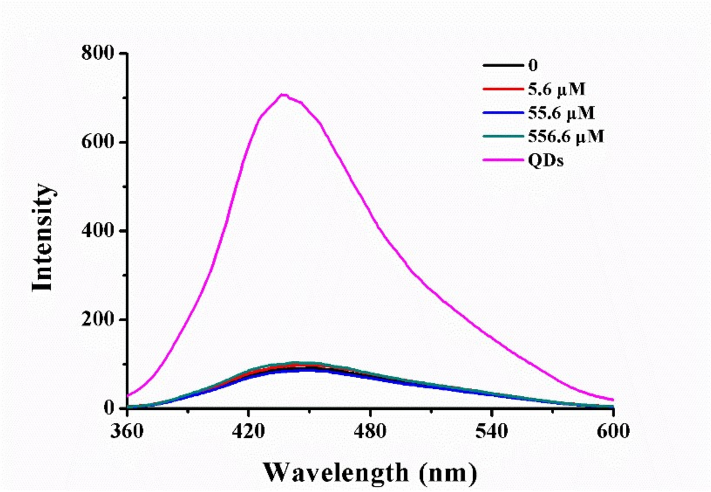

Figure S2. Fluorescence spectra of CQDs-Fe^{3+} system with different concentrations of gentamicin (0, 5.6 μM, 55.6 μM and 556.6 μM). And pink line was fluorescence spectrum of CQDs as a comparison.

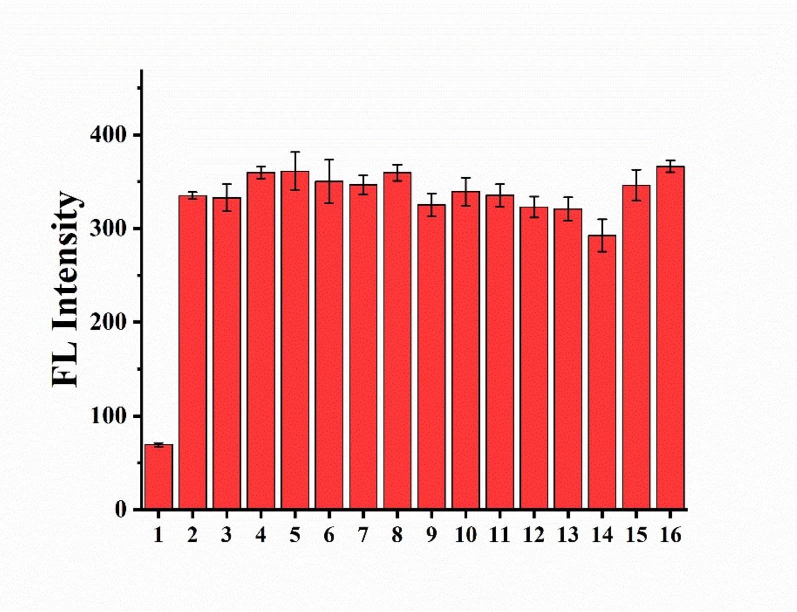

Figure S3. Anti-interference capability of CQDs-AuNPs system for gentamicin. 1 was FL intensity of CQDs-AuNPs system. 2-15 were amoxicillin, erythromycin, clarithromycin, ampicillin, penicillin, roxithromycin, norfloxacin, ofloxacin, thiamphenicol, clindamycin, trimethoprim, sulfapyridine, tetracycline, cefodizime sodium mixed with gentamicin. 16 was gentamicin. The concentrations of norfloxacin, ofloxacin, and methotrexate were 5 μM. The concentration of clindamycin was 1μM. The concentration of gentamicin was 200 nM. The other antibiotic concentrations were 50 μM.

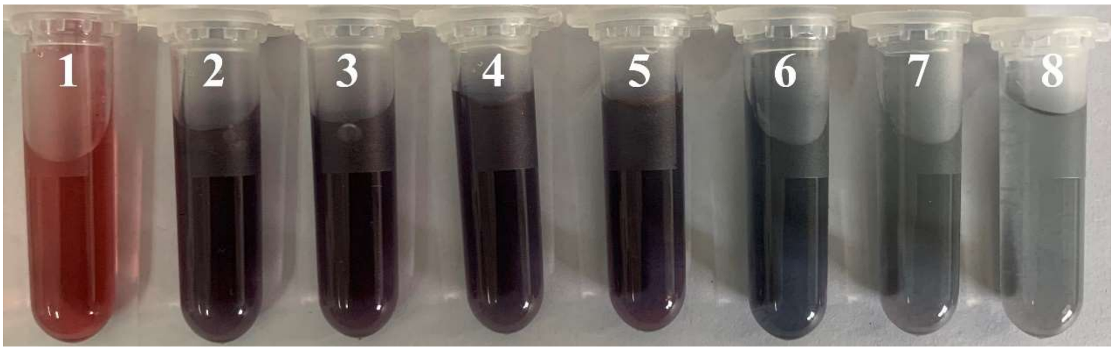

Figure S4. Colorimetric photograph of after the addition of various concentrations of gentamicin in watermelon juice samples. The final concentrations of 1-8 respectively were 0, 0.56 nM, 2.80 nM, 5.56 nM, 27.80 nM, 55.56 nM, 555.56 nM and 5.56μM.

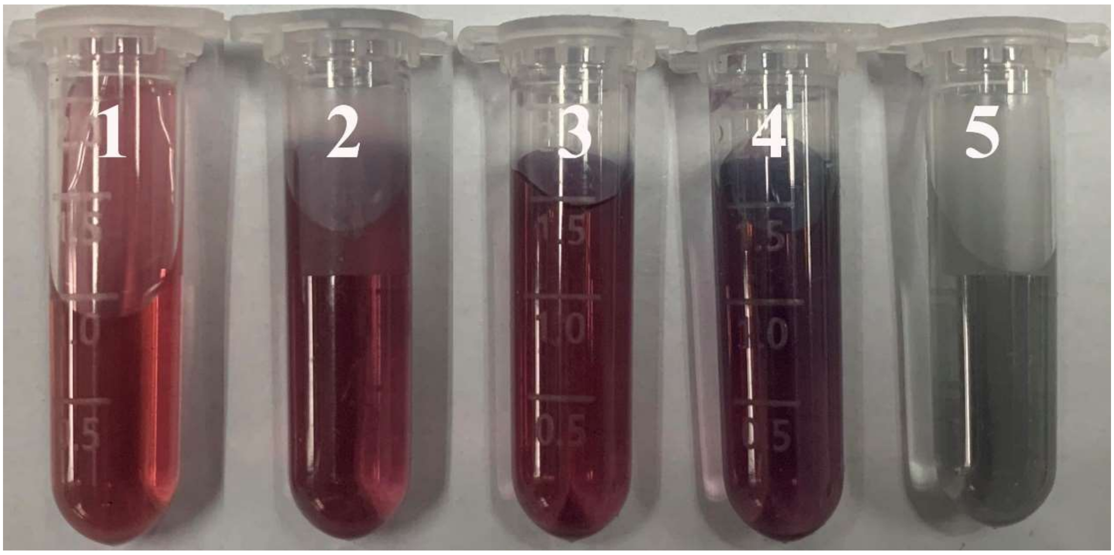

Figure S5. Colorimetric photograph of after the addition of various concentrations of gentamicin in cucumber juice samples. The final concentrations of 1-5 respectively were 0, 0.56 nM, 5.56 nM, 55.56 nM and 555.56 nM.
